# Changes in the Care of Neurological Diseases During the First Wave of the COVID-19 Pandemic: A Single Private Center Study in Argentina

**DOI:** 10.3389/fneur.2021.613838

**Published:** 2021-09-03

**Authors:** Ismael L. Calandri, Maximiliano A. Hawkes, Mariano Marrodan, Sebastián F. Ameriso, Jorge Correale, Ricardo F. Allegri

**Affiliations:** ^1^Departamento de Neurología, Fleni, Buenos Aires, Argentina; ^2^Department of Neurological Sciences, University of Nebraska Medical Center, Omaha, NE, United States

**Keywords:** neurology, COVID-19, Latin America, neurological care, telemedicine, lockdown

## Abstract

**Introduction:** Healthcare systems are struggling to cope with the rapid evolution of the COVID-19 pandemic. In Argentina, the pandemic is advancing despite prolonged lockdown measures. We aim to analyze the impact of the easing of lockdown measures in the number of visits to the emergency department (ED), and outpatient consultations (OC) to a tertiary neurological center.

**Methods:** We compared the number of ED visits with the social mobility overtime. We also compared the number of OC, and the geographic distribution of patients' addresses between 2019 and 2020.

**Results:** ED visits decreased 48.33% (*n* = 14,697 in 2019 vs. *n* = 7,595 in 2020). At the beginning of the lockdown, the social mobility decreased in pharmacies/groceries, and workplaces, along with a reduction in the number of ED visits. With the easing of lockdown restrictions, the social mobility decreased in residential places, slightly increased in workplaces and almost return to normal in pharmacies/groceries. Variations in ED visits correlate better with social mobility in workplaces (coef. =0.75, *p* < 0.001) than in groceries/pharmacies (coef. =0.68, *p* < 0.001). OC decreased 43%. Fourteen percent of OC were tele consults. This was associated with an increase of the geographical area of influence of our center (standard distance of 109 km in 2019 and 127 km in 2020).

**Conclusions:** Despite an increase in social mobility, the number of ED visits and OC to an Argentinian tertiary neurological center remain worrisomely low. The pandemic catalyzed the introduction of telemedicine in our country. This has also allowed patients from distant zones to gain access to specialized neurological care.

## Introduction

The COVID-19 pandemic has challenged healthcare systems worldwide (1). Social distancing, frequent hand sanitation, contact tracing, widespread testing, and the use of face masks are generally accepted strategies to stop the spread of the disease (2). Yet, the implementation of lockdowns, particularly their extent and duration, is still a matter of debate (3). This is particularly relevant in low- and middle-income countries where the negative impact on the economy may carry worse consequences than the pandemic itself (4). Additionally, strict lockdown measures may have unfavorable consequences for the mental health of the population and treatment of chronic diseases.

Argentina implemented a strict nationwide lockdown on March 20, 2020, with only 128 COVID-19 cases and 3 COVID-19-related deaths reported (5). Mass media, health authorities, and the medical community strongly recommended avoiding “non-urgent” contacts with the healthcare system in anticipation of a surge in COVID-19-related consults (5). Then, we reported a dramatic decline in the number of consults to the emergency department (ED) and outpatients clinics (OCs) of a tertiary neurological center (5). We emphasized the importance of tailoring the lockdown measures to particular epidemiological situations and providing appropriate medical care for non-COVID-19 medical conditions (5). Lockdown measures were partially relaxed on April 27, 2020. Some commercial and outdoor activities were allowed, while public or private events with more than 10 people, public transportation for non-essential workers, touristic activities, and attendance to cinemas, theaters, clubs, and cultural centers remained prohibited, despite distressing social and economic situations (6).

In this article, we aim to analyze the impact of easing lockdown measures on the number of visits for acute neurological conditions during the first wave of the pandemic. We also explored the impact of the first wave of the COVID-19 pandemic in the number and modality of neurological outpatient visits.

## Methods

### Study Design

We compared the number of visits to the ED of a tertiary neurological center with the mobility of the population from March 1 to August 31, 2020.

The number of visits was evaluated by reviewing the institutional electronic administrative records. A process of consolidation and data wrangling ensures data reliability. Administrative data of a medical consultation, the department where it was performed, and clinical data from the EMR are automatically integrated to avoid redundancies. Lastly, data are reviewed by a medical audit team.

We used the Google Mobility Index to evaluate the mobility of the population in the Buenos Aires Metropolitan Area. We analyzed three areas: pharmacies/groceries, residential places, and workplaces (7). The Buenos Aires Metropolitan Area (3,830 km^2^, 13,641,973 inhabitants) includes the Autonomous City of Buenos Aires and 24 adjacent districts of the Province of Buenos Aires (8).

The Google Mobility Index collects data from cell phones with Android operating systems or Google applications (e.g., Google Maps) to generate georeferenced information about people's movement trends over time by geography. The data are anonymized, aggregated by specific regions, and open access. The Google Mobility Index may be particularly useful in Argentina since 92% of cell phone devices use Android software (9). Furthermore, it has been successfully used to study the excess of cerebrovascular mortality during the COVID-19 pandemic (10).

We present the changes in social mobility and ED visits as the percentage reduction of the Google Mobility Index and the number of visits to the ED from 2019 to 2020, respectively. To avoid inaccuracies in the number of visits to the ED, as well as social mobility caused by weekends and holidays, we matched weekdays in 2019 and 2020. We also compared the number of outpatient visits, and the geographic distribution of patient's addresses between March 1, 2020, and August 31, 2020, with the same period in 2019. The geographical distribution of patient's addresses is shown on a map as points and point densities. To graphically emphasize possible changes in the number of outpatient visits of patients who live outside the Buenos Aires Metropolitan Area, we used a natural break classifier to determine the most appropriate scale. The borders of all Argentinian provinces have remained closed since the beginning of lockdown. Hence, all outpatient visits with an address outside the Great Buenos Aires Metropolitan Area were done with telemedicine.

This research was approved by the Institutional Ethics Committee.

### Statistical Analysis

A Pearson correlation was used to assess the synchrony of the mobility of the population and the number of ED consults overtime. The results are presented as correlation coefficients. A *p*-value < 0.01 was considered significant.

We also calculated the standard distance between our center and the addresses of patients who consulted in 2019 and compared it with 2020. Data analysis was performed with Python Software, version 3.8. Data wrangling was performed with Pandas (11) and NumPy (12) packages. The statistical analysis was performed with SciPy (13) and charts were made with Matplotlib package (14). The geographical analysis was performed with ArcGIS Desktop 10.8 (ESRI) software (15). For graphical representation and analysis, we used WGS 1984 Web Mercator (Auxiliary Sphere) spatial reference.

## Results

Our institution is a 113-bed tertiary academic center located in Buenos Aires City, Argentina (16). It is exclusively committed to the attention of neurological and neurosurgical diseases. Although our center is a national and regional referral center, its main influence is the Buenos Aires Metropolitan Area.

The healthcare system in Argentina is segmented and heterogeneous. It is based on the public provision of healthcare for every habitant. Additionally, 52% of people are covered by worker's organizations, 9% by private health insurances, and 8.3% by government-funded social insurance for the retired population “PAMI” (17). The majority of our patients have private or worker's organizations health insurance. Before the beginning of the pandemic, no health insurance covered telemedicine consults. Hence, while there was an operational telemedicine service, only sporadic consults were performed.

### Impact of Lockdown Measures Easing in the Number of Visits to the ED

We analyzed *n* = 92,534 administrative records from the ED, *n* = 71,917 from 2019, and *n* = 20,617 from 2020. Compared with the same period of 2019, the number of visits to the ED during March–August 2020 decreased 48.33% (*n* = 14,697 in 2019 vs. *n* = 7,595 in 2020). The most frequent reason for ED consultation was headache (2019 = 53% and 2020 = 57%) and the most frequent reason for hospital admission was ischemic stroke (2019 = 28%, 2020 = 33%). There were no significant differences in the reason for ED consultation between 2019 and 2020 (*p* = 0.17).

**Figure 1:**
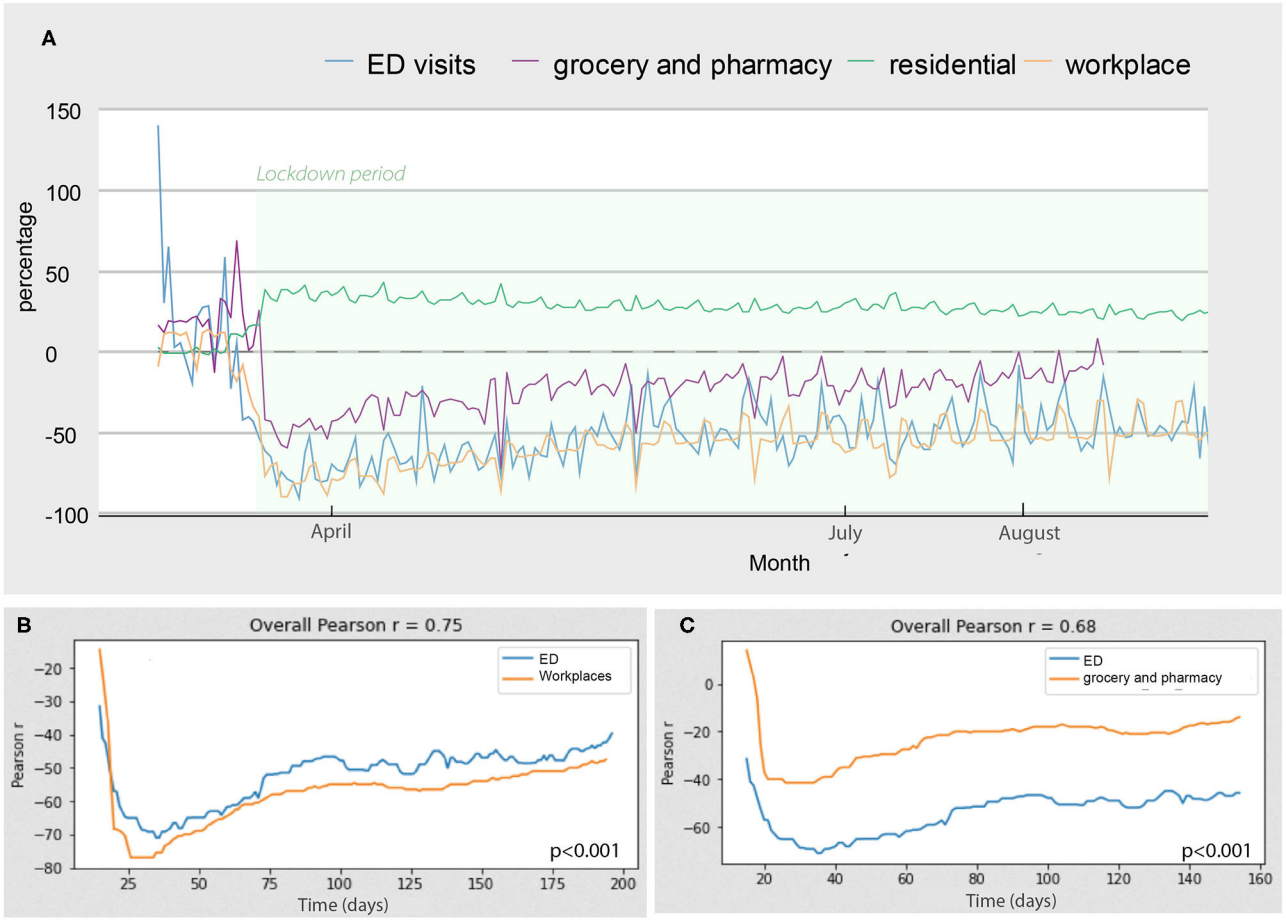
Evolution of the number of ED consults and social mobility. Changes in social mobility and number of ED visits over time. **(A)** The lines represent the percentage of change compared to the same period of 2019. The correlation plots show the correlation between the number of ED consults and social mobility in workplaces **(B)** and between the number of ED consults and social mobility in groceries and pharmacies **(C)**.

Figure 1 shows the comparison between the number of visits to the ED and social mobility over time. At the beginning of the lockdown, social mobility increased in residential areas and decreased in pharmacies/groceries and workplaces. This correlates with a sharp reduction in the number of visits to the ED (Figure 1A). As lockdown measures relaxed, we observed the opposite. A correlation analysis showed that the number of ED visits had a small increase, similar to the increased social mobility in workplaces (correlation coefficient 0.75, *p* < 0.001, Figure 1B). However, the social mobility in groceries/pharmacies increased significantly more than the number of ED visits (correlation coefficient 0.68 *p* < 0.001, Figure 1C).

### Impact of the Pandemic on In-hospital Mortality

Our center recorded 21 deaths and 1,712 admissions in 2019, and 30 deaths in 1,278 admissions during 2020. The median monthly mortality was 2.5 in 2019 compared to 5.5 in 2020. Although numerically higher, the difference is not statistically significant (*p* = 0.058). In 2020, the causes of death were stroke (*n* = 4), status epilepticus (*n* = 2), complications of chronic neurological diseases (*n* = 11), rupture of abdominal aortic aneurysm (*n* = 1), and complications of brain tumors (*n* = 12). In 2019, the causes of death were stroke (*n* = 3), complications of chronic neurological diseases (*n* = 4), status epilepticus (*n* = 1), and complications of cancer/brain tumors (*n* = 12).

### Impact of the Pandemic in the Number and Modality of Outpatient Visits

A total of 141,772 outpatients visits were registered during March–August 2019. This number decreased to 64,343 in 2020 (43% decrease). The reduction in the number of consults peaked in April (almost 90%), in hand with the beginning of strict lockdown measures. Table 1 shows the monthly percentage reduction in outpatient visits according to neurological subspecialty. A total of 9,014 outpatient visits were teleconsults. This represents 14% of all outpatient consultations performed during the same period of time. Most teleconsults were follow-up visits for chronic neurological diseases (78%), and the remainder were first-time evaluations (22%) (**Figure 3**). The geographical analysis of the outpatient's addresses reveals a significant increase in the area of influence of our center (**Figure 3**). The standard distance of patient's addresses to our medical center increased from 109 km in 2019 to 127 km in 2020 (**Figure 3**).

**Table 1:** Percentage reduction in outpatient visits according to month and neurological subspecialty.

**Department**	**March**		**April**		**May**		**June**		**July**		**August**	
	**2019** **(***n***)**	**2020 (%)**	**2019** **(***n***)**	**2020 (%)**	**2019** **(***n***)**	**2020 (%)**	**2019** **(***n***)**	**2020 (%)**	**2019** **(***n***)**	**2020 (%)**	**2019** **(***n***)**	**2020 (%)**
Endovascular Neurosurgery	72	−15.3%	91	−85.7%	76	−38.2%	82	−47.6%	95	−44.2%	92	−40.2%
Cardiology	846	−24.5%	919	−89.8%	997	−54.1%	863	−26.7%	912	−21.1%	1,027	−31.5%
Headache and Pain	1,016	−42.9%	1,048	−88.4%	1,270	−59.7%	983	−24.6%	1,079	−25.2%	1,197	−30.6%
Internal Medicine	357	−33.3%	405	−88.1%	384	−56.5%	368	−35.3%	451	−36.1%	402	−28.4%
Electrophysiology	16	−12.5%	33	−78.8%	25	−36.0%	21	−61.9%	25	−84.0%	32	−65.6%
Neuromuscular Disorders	222	17.1%	218	−61.9%	252	−21.4%	252	6.7%	303	−2.0%	261	18.0%
Epilepsy	249	−19.7%	284	−89.8%	312	−51.6%	262	−20.2%	302	−30.5%	301	−35.2%
EEG^*^ Video-Telemetry	172	−23.3%	166	−88.6%	168	−38.1%	151	−30.5%	199	−39.7%	210	−41.9%
Multiple Sclerosis	411	−21.4%	444	−86.0%	424	−24.8%	407	2.7%	531	−20.2%	417	−4.1%
Sleep medicine	216	−91.7%	206	−100%	204	−100%	145	−78.0%	158	−38.7%	212	−15.8%
Movement Disorders	478	−30.1%	528	−94.3%	541	−50.3%	387	−11.1%	484	−36.4%	565	−34.9%
Neuro-Ophtalmology	240	−37.9%	282	−92.2%	319	−65.8%	174	16.7%	309	−44.0%	263	−19.4%
Neurosurgery (adults)	600	−26.2%	607	−85.0%	731	−64.6%	636	−36.2%	572	−28.7%	728	−39.7%
Pediatric Neurosurgery	189	−16.4%	188	−94.1%	228	−71.1%	201	−54.7%	218	−52.3%	238	−43.7%
Neuroendocrinology	163	20.2%	264	−92.8%	311	−45.3%	269	−21.6%	307	−28.7%	327	−42.5%
Neurophysiology	18	−66.7%	23	−30.4%	28	−85.7%	16	−68.8%	25	−96.0%	22	−72.7%
Stroke	612	−33.5%	674	−90.2%	697	−52.8%	738	−33.5%	838	−46.9%	796	−38.8%
General Neurology	1,607	−35.7%	1,518	−92.0%	1,673	−52.4%	1,530	−40.3%	1,769	−47.9%	1,860	−45.3%
Cognitive Neurology	754	−32.5%	671	−90.8%	754	−59.5%	583	−11.8%	741	−40.2%	809	−31.8%
Pediatric Neurology	739	−42.5%	878	−92.3%	916	−45.2%	886	−22.5%	1,117	−30.9%	1,004	−21.9%
Neuro-Oncology	415	−12.9%	492	−31.5%	473	−18.2%	438	−4.6%	523	−24.9%	481	−10.0%
Neuro-Orthopedic	510	−34.7%	438	−94.5%	524	−70.6%	442	−50.2%	478	−61.1%	475	−42.3%
Neuro-Otology	360	−42.8%	458	−91.9%	432	−59.7%	378	−23.5%	433	−25.6%	516	−31.6%
Psychiatry	546	−49.3%	472	−96.0%	535	−63.7%	495	−34.1%	513	−37.4%	612	−39.5%
Total	10,808	−30.85%	11,307	−87.69%	12,274	−52.52%	10,707	−25.37%	12,382	−34.24%	12,847	−31.46%
											70,325	−42.67%

* *Electroencephalogram*.

## Discussion

The consequences of the COVID-19 pandemic exceed the COVID-19 disease itself (18). During the peak of the first wave of the pandemic, Europe and had an increase in non-COVID-19-related deaths of 14 and 25%, respectively (19). There are several potential explanations for this: (1) a proportion of COVID-19-related deaths have not been counted as such, (2) avoidable non-COVID-19-related diseases that could not be properly treated because of the saturation of healthcare systems, (3) decompensation of chronic medical conditions due to in adequate medical care, (4) late or no consultation to the ED due to fear of contracting COVID-19 and/or wrong messages from mass media, healthcare authorities, and medical community (5, 19, 20).

Several studies have reported a dramatic drop in the number of visits to the ED for serious and urgent medical conditions such as heart attack and stroke (20). During the beginning of the lockdown, we reported that the number of admissions for acute ischemic stroke and transient ischemic attacks decreased 50 and 80%, respectively. Also, the number of patients who consulted the ED or OC with MS relapses and seizures decreased 50 and 83%, respectively (5).

The findings of the present study show that, 5 months after the beginning of pandemic, and despite partial relaxation of lockdown measures, the number of emergent and outpatient visits to a tertiary neurological Argentinian center remained low in comparison to the increase in social mobility in areas such as pharmacies and groceries. Whether our results can be extrapolated to the entire Argentinian healthcare system is difficult to ascertain due to the paucity of published data, lack of specific public statistics, and the single-center design of our study. However, as all medical centers were requested to remain fully available for a potential surge in COVID-19 cases, most outpatient visits were not allowed, and the message to the population was to avoid “unnecessary” or “non-urgent” medical consultations; we believe that our data may reflect the trend in a significant proportion of the Argentinian healthcare facilities.

Social mobility in pharmacies/groceries is common in nearby shops. Hence, this was unlikely affected by the unavailability of public transportation. The decreased mobility in residential places also means that social mobility increased (Figure 1). The social mobility in workplaces marginally increased compared to the strict lockdown period but remained low compared to the increased social mobility in pharmacies and groceries. This can be explained by the fact that most non-essential activities were prohibited by law during the study time frame. Also, public transportation was available only for essential workers, limiting the mobility of the majority of workers and patients. Altogether, our data suggest that continuous efforts are still needed to improve and refine the communication to the population concerning the importance of continuing regular medical check-ups and visiting the ED when acute symptoms develop. Non-COVID-19 diseases still occur, matter, and many can be properly prevented and treated, avoiding irreversible neurological sequelae in many cases. This issue must be clearly explained to people. Patients who need regular infusions for chronic diseases, such as immunosuppressive treatments for MS, should be particularly counseled about the dangers of discontinuation of their treatments. Although, at the beginning of the pandemic, it was suggested to defer the time of administration of some immunosuppressive drugs, now with the evidence that the COVID-19 pandemic will most likely remain for a long period, these considerations are under review (21).

**Figure 2:**
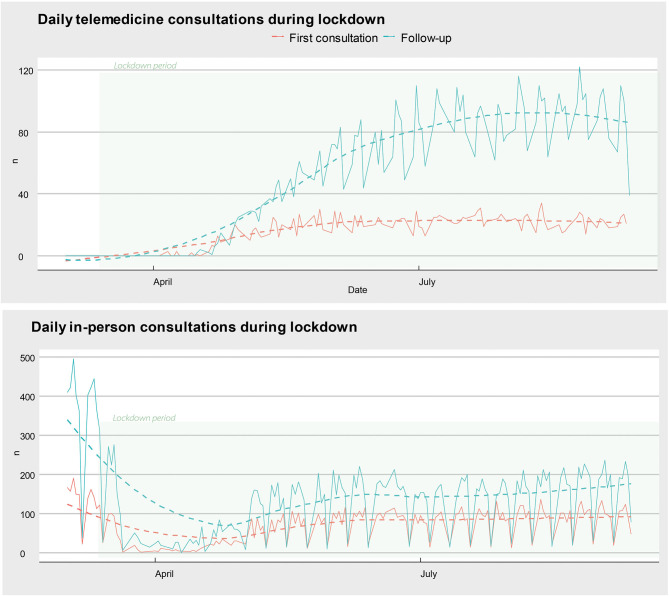
Evolution of telemedicine and in-person consults during the lockdown. The number of first-time (red) and follow-up (blue) visits over time. The dashed lines represent the time trend in a general linear model. Of note, telemedicine visits increase in early April, shortly after the beginning of the lockdown mandate (March 20).

**Figure 3:**
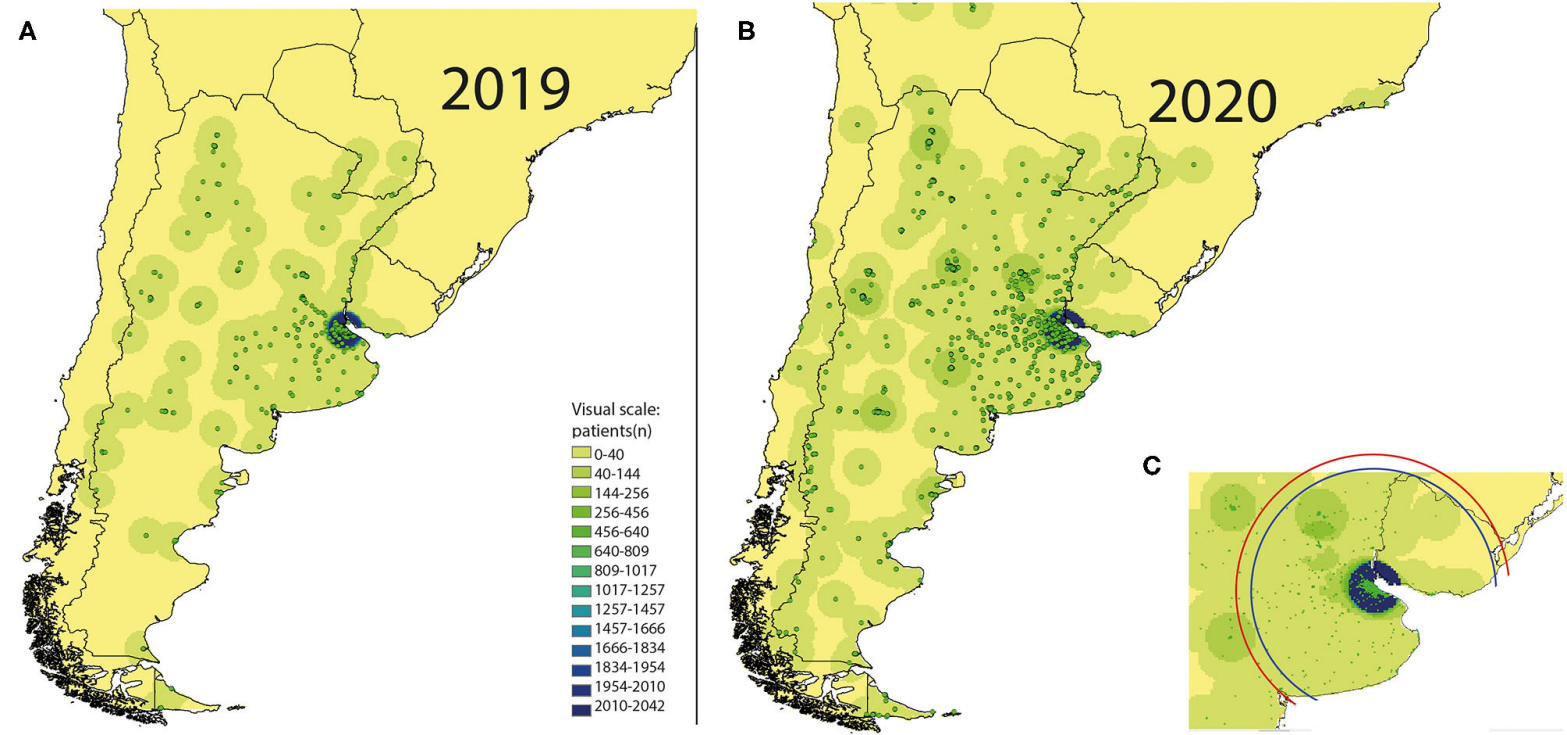
Changes in the geographical area of influence of our center due to the introduction of telemedicine. The points and point densities represent the number of patients according to the geographical location of their addresses. Compared to 2019 **(A)**, in 2020 **(B)**, more patients from distant areas of Argentina and bordering countries had access to our center. During the study time frame, all the national and international borders remain closed. Thus, the observed changes can be attributed to the introduction of telemedicine. **(C)** shows the standard distance measured from the patient's addresses to our center in 2019 (blue circle = 109 km) and 2020 (red circle = 127 km).

We also found that despite a dramatic drop in the total number of outpatient visits, the use of telemedicine increased substantially, particularly for follow-up visits (Figure 2). Remarkably, the pandemic catalyzed the introduction of telemedicine in our country, mainly because of improvements in the coverage of healthcare insurances. These advances may be due to a combination of increased social demand and public health needs. In this context, teleconsults are particularly useful to facilitate the access of patients with chronic neurological conditions at high risk of developing severe COVID-19 disease (e.g., patients with MS on chronic immunosuppressants) to regular medical follow-up visits (22). The descriptive analysis of the maps in Figure 3 suggests that the introduction of telemedicine for outpatient visits has expanded the area of influence of our center, allowing patients from distant areas of the country and bordering countries to gain access to specialized neurological care. This is particularly important in Latin America because the number of neurologists per 100,000 inhabitants is low in many regions and they tend to concentrate in big cities (23, 24).

Developing efficient technologic tools and providing massive access to telemedicine has been challenging worldwide. While in some countries telemedicine could be implemented efficiently without delays, significant difficulties and disparities persist in several areas (25–28). This reinforces the importance and continues the need for multidisciplinary work to develop more efficient communication tools for patients and healthcare providers and to generalize access to the internet, particularly in underserved areas.

The main strength of this investigation is the detailed comparison of neurological service use during the first wave of the pandemic period to a control period for all types of neurological disorders, and comparisons of service use to the changes in social mobility. This study also has limitations worth mentioning. First, we report data from a single tertiary center, limiting its generalization. Second, it contemplates the effects of the lockdown measures in Argentina. Hence, data cannot be extrapolated to other countries with different social, demographic, economic, and healthcare structures. Third, we report data of a private hospital: the majority of patients serviced had extra insurance in addition to the public provision of healthcare. Thus, the results may not be representative of the total healthcare service use of the country due to a selection bias. Fourth, we only evaluated the first wave of the pandemic. Fifth, our data set does not allow us to evaluate potential morbidity and mortality of patients who did not seek medical attention or consulted to another center. A population-based registry and publicly available official data are needed to further explore this issue.

## Conclusions

In Argentina, 5 months after the beginning of the pandemic, and despite an increase in social mobility, the number of visits to the ED and OC of a tertiary neurological center remains worrisomely low. The pandemic catalyzed the introduction of telemedicine in our country. This has also allowed patients from distant zones to gain access to specialized neurological care.

## Data Availability Statement

The raw data supporting the conclusions of this article will be made available by the authors, without undue reservation.

## Author Contributions

IC and MH contributed equally to the manuscript. IC, MH, and MM recolect data, interpret results, and write the manuscript. IC perfomed the statistical analysis. JC, SA, and RA provided original ideas, review manuscript, and add interpretation. All authors contributed to the article and approved the submitted version.

## Conflict of Interest

The authors declare that the research was conducted in the absence of any commercial or financial relationships that could be construed as a potential conflict of interest.

## Publisher's Note

All claims expressed in this article are solely those of the authors and do not necessarily represent those of their affiliated organizations, or those of the publisher, the editors and the reviewers. Any product that may be evaluated in this article, or claim that may be made by its manufacturer, is not guaranteed or endorsed by the publisher.
